# Retention of knowledge and skills in pediatric basic life support amongst pediatricians

**DOI:** 10.1007/s00431-018-3161-7

**Published:** 2018-05-07

**Authors:** Mathijs Binkhorst, Michelle Coopmans, Jos M. T. Draaisma, Petra Bot, Marije Hogeveen

**Affiliations:** 1 Department of Neonatology (804), Radboud University Medical Centre Amalia Children’s Hospital, P.O. Box 9101, 6500 HB Nijmegen, The Netherlands; 2 Department of Pediatrics, Radboud University Medical Centre Amalia Children’s Hospital, Nijmegen, The Netherlands

**Keywords:** Cardiopulmonary resuscitation, Child, Clinical competence, Basic cardiac life support, Pediatrics, Retention

## Abstract

Retention of resuscitation skills is usually assessed at a predefined moment, which enables participants to prepare themselves, possibly introducing bias. In this multicenter study, we evaluated the retention of knowledge and skills in pediatric basic life support (PBLS) amongst 58 pediatricians and pediatric residents with an *unannounced* examination. Practical PBLS skills were assessed with a validated scoring instrument, theoretical knowledge with a 10-item multiple-choice test (MCQ). Participants self-assessed their PBLS capabilities using five-point Likert scales. Background data were collected with a questionnaire. Of our participants, 21% passed the practical PBLS exam: 29% failed on compressions/ventilations, 31% on other parts of the algorithm, 19% on both. Sixty-nine percent passed the theoretical test. Participants who more recently completed a PBLS course performed significantly better on the MCQ (*p* = 0.03). This association was less clear-cut for performance on the practical exam (*p* = 0.11). Older, attending pediatricians with more years of experience in pediatrics performed less well than their younger colleagues (*p* < 0.05). Fifty-one percent of the participants considered themselves competent in PBLS. No correlation was found between self-assessed PBLS capabilities and actual performance on the practical exam (*p* = 0.25).

*Conclusion*: Retention of PBLS skills appears to be poor amongst pediatricians and residents, whereas PBLS knowledge is retained somewhat better.

**Table:** 

**What is Known:** • *Pediatricians and pediatric residents are not always competent in pediatric basic life support (PBLS) in daily practice. Poor retention of skills supposedly accounts for this incompetence. Without regular exposure, resuscitation skills usually deteriorate within 3 to 6 months after training*. • *Examination of resuscitation skills usually takes place after training. Also, in most studies evaluating retention of skills, participants are tested at a predefined moment. Inasmuch as participants are able to prepare themselves, these assessments do not reflect the* ad hoc *resuscitation capabilities of pediatricians and residents.*	
**What is New:** • *In this study, pediatricians and pediatric residents had to complete an unannounced PBLS exam at variable time intervals from last certification. Retention of PBLS skills was rather poor (pass rate 21%).* • *The PBLS skills of older, attending pediatricians with many working years in pediatrics appeared to be inferior to those of their younger colleagues.*

**Electronic supplementary material:**

The online version of this article (10.1007/s00431-018-3161-7) contains supplementary material, which is available to authorized users.

## Introduction

Cardiac arrest is an uncommon event in children (9 per 100.000 children per year) often with a poor outcome [3]. An overall survival rate of 6% has been found in children with cardiac arrest in out-of-hospital settings [3]. Healthcare professionals who work with children are expected to be competent in pediatric basic life support (PBLS) according to national guidelines, which are usually based on the European Resuscitation Council (ERC) or American Heart Association (AHA) guidelines [2, 16]. In order to acquire and consolidate this competency, pediatricians and pediatric residents follow resuscitation courses, including PBLS [11].

Based on the literature, pediatric professionals are not fully competent in providing PBLS [28]. Although poor *acquisition* of skills may be the problem, poor *retention* of skills is probably the main reason underlying this incompetency [13, 14, 19–21, 26–28]. Studies have shown that resuscitation skills, when used infrequently, deteriorate within 3 to 6 months after training and that knowledge is retained longer than skills [5, 7, 22, 27]. In addition, (pediatric) residents and junior doctors have reported that they do not feel confident about performing (P)BLS during an actual emergency situation [6, 10, 30]. This lack of confidence could lead to a delay in incipience of resuscitation [6].

Since PBLS skills are usually examined immediately after training, the large majority of participants pass these exams (Dutch Foundation for the Emergency Medical Care of Children, personal communication). In previous studies investigating retention of skills, participants were almost always retested at a predefined moment. We believe that this practice of examining at a specified moment (after training) is not representative of the pediatricians’ ability to resuscitate a child ad hoc. Therefore, we sought to determine the retention of PBLS knowledge and skills amongst pediatricians and pediatric residents with an *unannounced* PBLS examination. Factors potentially influencing retention of PBLS skills were also assessed.

## Materials and methods

### Participants

In view of the time frame of our study (~ 3 months), the anticipated logistic challenges, and number of participants in related studies, we aimed to include a convenience sample of at least 50 participants. Because a high refusal rate was foreseen considering our study design with unannounced visits, we addressed a large surplus of hospitals. Pediatricians and pediatric residents of 8 academic and 17 general Dutch hospitals were invited to participate in this study, which took place in 2016. The Institutional Review Board waived formal approval. Hospitals were included only when the entire pediatric department gave informed consent in order to prevent selection bias. When consent was obtained, the department was notified that we would visit the hospital unexpectedly in the coming year. During the visit, written informed consent was obtained from all individual participants.

### Data collection

All participants completed the PBLS exam in the order presented below. To minimize this risk of test results influencing each other, participants were not informed about their results before completion of the entire exam. The complete examination procedure lasted 8–10 min per participant. The examiners of the practical exam and multiple-choice test (MCQ) were blinded to the results of the questionnaire and self-assessment.

#### Assessment of skills

All participants performed a standard PBLS scenario (Table 1) on the same pediatric resuscitation manikin (Resusci® Junior Basic, Laerdal Benelux, The Netherlands), taken along by the investigators. We used a manikin without skillmeter, considering the great number of non-recording manikins in active use (in The Netherlands) and because our scoring instrument has been validated for non-recording manikins. Scenarios lasted ~ 2 min and included initial approach, assessment, call for assistance, and rescue breaths (~ 1 min), followed by 4 cycles of compressions and ventilations (~ 1 min). The use of an automated external defibrillator (AED) was not tested, for it would have required too much time during our short, unannounced visits. Moreover, AED use is not examined in conjunction with the PBLS algorithm during the European Pediatric Advanced Life Support (EPALS) course, and our validated scoring instrument does not incorporate items to score AED use. Pediatricians must be able to perform PBLS without equipment; ventilations were therefore delivered mouth-to-mouth. As pediatricians are professionals trained to use a compression-ventilation ratio of 15:2 in children, we only approved this ratio. All participants were assessed by the same two examiners, one to observe technique and one to record time intervals.

**Table 1:** Situation, sequence, and scenario for the unannounced examination of pediatric basic life support skills

Situation	
• Two investigators (MB, MC) enter the hospital • Secretary guides both investigators to a consulting room • Pediatricians and pediatric residents ignorant of the investigators’ arrival • Preparation of the manikin (Resusci® Junior Basic) • Preparation of other test materials (e.g., scoring forms, questionnaires, stopwatch)	
Sequence	
• Secretary summons pediatricians and residents consecutively to the consulting room • Test procedure and scenario are explained to the participant • Participant signs informed consent form • Participant performs practical PBLS exam • Participant completes MCQ, questionnaire, and self-assessment • Participant receives feedback on his/her performance • Participant is instructed not to notify his/her colleagues about our presence	
Scenario	
• Participant is instructed to perform PBLS according to the Dutch guideline^1^ • Participant is instructed to work through the entire algorithm, including chest compressions • Participant is instructed to continue until investigators say “ambulance has arrived” • Scenario involves an approximately 8-year-old boy found unconscious on the pavement • No foreign body airway obstruction • No trauma • No witnessed sudden collapse • Investigators serve as fictitious bystanders • No cues or suggestions given by the investigators • Use of an AED not tested in this scenario • All participants tested individually	

*PBLS* pediatric basic life support, *MCQ* multiple-choice test, *AED* automated external defibrillator

^1^The PBLS guideline of the Dutch Resuscitation Council is equivalent to the ERC guideline

The participants’ performance was assessed with the validated Modified Berden Score (Table 2). This scoring instrument is firmly based on the 2015 ERC algorithm for PBLS [16]. Its validation process and use in conjunction with a low-fidelity manikin are elaborately discussed in the original article (Binkhorst et al.: Modified Berden Score for pediatric basic life support assessment, submitted), the abstract of which is available online (Abstracts/Resuscitation 118S (2017) e8; 10.1016/j.resuscitation.2017.08.033). Minor, moderate, substantial, and fatal errors were assigned 5, 10, 15, and 20 penalty points, respectively. A maximum of 145 penalty points could be scored. The cutoff for a pass score was ≤ 15 penalty points [4]. Items 3c, 5, 6, and 7a were referred to as “compressions/ventilations,” the remaining items as “other parts of the algorithm.”

**Table 2:** Modified Berden Score for pediatric basic life support assessment

Task	Performance	Penalty points
1. Safe-stimulate-shout		
a. Ensure safe environment	Yes	0
	No	5
b. Assess responsiveness	Correct	0
	Incorrect	5
c. Shout for help	Yes	0
	No	5
2. Airway		
a. Chin lift	Correct	0
	Incorrect	5
3. Breathing and medical assistance		
a. Look-listen-feel	Correct	0
	Incorrect	5
b. Call emergency number and ask for AED	Yes	0
	Incomplete	5
	No	10
c. Five initial rescue breaths	0–1 inadequate	0
	2–4 inadequate	5
	5 inadequate/not done	10
4. Circulation		
a. Look for signs of life and/or check pulse	Correct	0
	Incorrect	5
5. Compressions		
a. Hand/finger placement	Correct	0
	Incorrect	5
b. Arm position	Correct	0
	Incorrect	5
c. Duration of last 2 × 15 compressions	≤ 11 s	15
	12–13 s	10
	14–15 s	5
	16–20 s	0
	21–25 s	10
	26–30 s	15
	≥ 31 s	20
d. Average compression depth	Correct	0
	Too deep	5
	Too shallow	10
e. Leaning	No	0
	> 10%	5
	> 50%	10
6. Breaths		
a. Tidal volume	0–1 inadequate	0
	2 inadequate	5
	3 inadequate	10
	4 inadequate	15
	5–6 inadequate	20
b. Duration of last 3 × 2 breaths	≤ 18 s	0
	19–25 s	5
	26–32 s	10
	≥ 33 s	15
7. Ratio and sequence		
a. Correct compression-breath ratio	Yes	0
	No	5
b. Correct sequence of tasks	Yes	0
	No	5
Total number of penalty points		

**Scoring instructions**

*General statements*

• This scoring instrument can be used for infants and children.

• In this scenario, a fictitious bystander is present to call the emergency number.

• The casualty in this scenario did not suffer (cervical) trauma.

• PBLS examinations last approximately 2 min: 1 min for tasks 1–4 and 1 min for four CPR cycles.

• Examinations should be carried out by two examiners: one to observe technique and one to record time.

• If a task is performed in a way not specified in these instructions, consensus must be reached between two examiners on how to score the task.

• For a detailed description of correct task performance, see the 2015 ERC guidelines (Maconochie et al.).

• ≤ 15 penalty points is a pass score; > 15 penalty points is a fail score.

*Task-related instructions*

1. a: The examinee should either say “safe environment” or inspect the environment visibly.

b: Responsiveness should be assessed with both verbal and physical stimuli.

c: The bystander should be requested to stay in the vicinity of the casualty.

2. a: Five penalty points are assigned when (1) fingers are not hooked behind the chin bone, but obviously impressed on the soft tissue between the chin bone and thyroid cartilage; (2) fingers are placed on the chin without lifting it; (3) the chin lift prior to the look-listen-feel procedure is incorrect, even though it is adequate during ventilations. One may briefly inspect the oral cavity and remove visible obstructions; 10 penalty points for a blind finger sweep.

3. a: This procedure is incorrect if it lasts > 10 s.

b: A request to call the emergency number is incomplete when (1) it is not stated that a child is being resuscitated, and (2) the bystander is not requested to look for an AED.

c: Breaths are inadequate when the manikin’s chest does not rise or too much air is inflated.

Five penalty points are allocated when > 5 initial rescue breaths are performed (usually done to correct for inadequate breaths). When the nose is not pinched during ventilations, but the manikin’s chest rises adequately, no penalty points have to be assigned.

4. a: This procedure is incorrect if it lasts > 10 s. The examinee should at least look briefly for body movements or say something like “not responding.” Only professionals (incl. interns, residents, and skilled nurses) are allowed to check the pulse.

5. a: Five penalty points are given when (1) hand(s) or fingers are not placed on the lower half of the sternum; (2) in a child, fingers clearly exert pressure on the rib cage. Hand placement should preferably be scored during the last 2 × 15 compressions.

b: Arms should be vertical and stretched. Arm position should preferably be scored during the last 2 × 15 compressions. Arm position is not scored in infants.

c: This is the length of time of the last 2 × 15 compressions (i.e., third and fourth cycle) combined.

For the sake of clarity: 16–20 s means 16.00–20.99 s.

d: This is scored based on the last 2 × 15 compressions.

e: This is scored based on the last 2 × 15 compressions.

6. a: Breaths are inadequate when the manikin’s chest does not rise or too much air is inflated.

b: This is the length of time of the last 3 × 2 breaths (i.e., second, third, and fourth cycle) combined.

A breathing interval starts as soon as the hand(s) or fingers are removed from the manikin’s chest and ends when the hand(s) or fingers are placed back on the chest to resume compressions.

7. a: This is scored based on the last 2 cycles. Five penalty points are assigned when extra breaths are performed to correct for inadequate ones. If inadequate cycle breaths are correctly compensated, penalty points are given for ratio and (possibly) duration of breathing, but not for tidal volume.

b: Deviation from the correct sequence of tasks results in five penalty points (once).

#### Theoretical test

Theoretical knowledge was tested with a 10-item MCQ based on the 2015 ERC guidelines [16]. Prior to the actual study, our MCQ was critically appraised and amended by four PBLS course instructors and three experts in test development. There was only one unequivocal answer to each question. One point was allocated to each correct answer, without correction for guessing. Accordingly, participants obtained a score between 0 and 10. Participants passed the MCQ when scoring ≥ 8, based on current examination practices of life support courses [15, 23].

#### Questionnaire

The following participant characteristics were collected: sex, age, specialization level (resident or attending), hospital type (academic or general), and years of experience in pediatrics. We determined whether these factors correlated with the resuscitation skills of pediatricians and residents. To evaluate retention of skills, the questionnaire also included questions on the frequency with which participants had attended PBLS courses, the exposure to real-life in-hospital (IHCA) and out-of-hospital cardiac arrest (OHCA) in children, and the time interval from last PBLS certification.

#### Self-assessment

Participants rated their own capabilities regarding the following eight items: airway opening, assessment of breathing, assessment of circulation, compressions, ventilations, AED use, PBLS knowledge, and overall competence in PBLS. Although AED use was not tested during skills assessment for practical reasons, we included it in the self-assessment (and MCQ), considering its importance in PBLS. For each item, a five-point Likert scale was used. The cutoff point for self-assessed competence was arbitrarily set at ≥ 32 points (≥ 80%) on the combined Likert scales, because 80% (as a mark) generally reflects “good performance.”

### Statistical analysis

Data are presented as mean (SD) and/or median with interquartile range (IQR), as appropriate. Participant characteristics and assessment scores were compared with Spearman’s rank correlation coefficients and Kruskal-Wallis one-way analysis of variance for ordinal variables and non-parametric distributed data. Statistical analyses were performed using SPSS version 22.0. A *p* value < 0.05 was considered statistically significant.

## Results

One of 8 academic and 5 of 17 general hospitals agreed to participate. Of the general hospitals, three were teaching and two were non-teaching. Further details are not provided to ensure the anonymity of the hospitals and participants. The majority of the hospitals declined participation. The main reasons were, as expected, a lack of time and the idea that our unannounced visit would interfere with their daily activities. Some hospitals indicated that they were already involved in (enough) other studies. Eventually, 58 pediatricians and residents were available for testing. Twenty-one percent passed the practical PBLS exam. The overall mean penalty score on the practical exam was 27.9 (SD 12.9), median 25.0 (IQR 20.0–36.3). Twenty-nine percent of the participants failed only on compressions/ventilations, 31% on other parts of the algorithm, and 19% on both. The mean penalty score for compressions/ventilations, with a maximum of 100 penalty points, was 14.5 (SD 11.1). The mean penalty score for the other parts of the algorithm, with a maximum of 45 penalty points, was 13.4 (SD 6.8). Cardiopulmonary resuscitation (CPR) was not performed according to the correct compression-ventilation ratio of 15:2 by 29% of the participants. Most penalty points were scored for “ratio and sequence,” with a mean of 5.5 (SD 3.2) on a maximum of 10 penalty points. Fewest penalty points were scored for “breaths,” with a mean of 4.0 (SD 4.4) on a maximum of 35 penalty points.

The theoretical test had a pass rate of 69%, with an overall mean score of 7.6 (SD 1.9), median 8.0 (IQR 7.0–9.0). Only 19% of the participants passed both the theoretical and practical exam. A correlation was found between the practical and theoretical exam: a higher penalty score correlated with a lower score on the MCQ (Spearman’s rho − 0.32, *p* = 0.01).

**Fig. 1:**
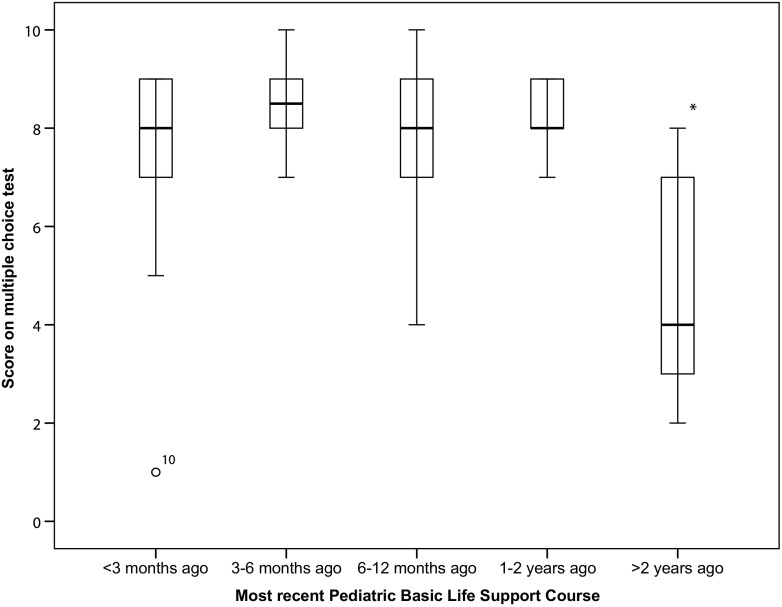
Association between time interval since last PBLS course and theoretical test result. * Significantly different (*p* < 0.05) compared to last PBLS course < 2 years ago

**Fig. 2:**
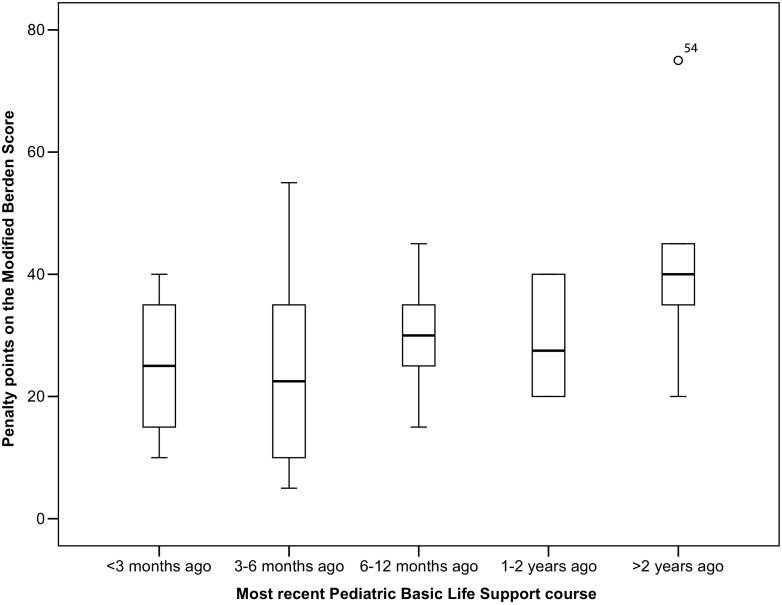
Association between time interval since last PBLS course and practical test result

Participants who more recently followed PBLS courses performed significantly better on the MCQ than those who followed their last course > 2 years ago (*p* = 0.03) (Fig. 1). The association between time since last PBLS course and performance on the practical exam was not statistically significant (*p* = 0.11) (Fig. 2).

**Fig. 3:**
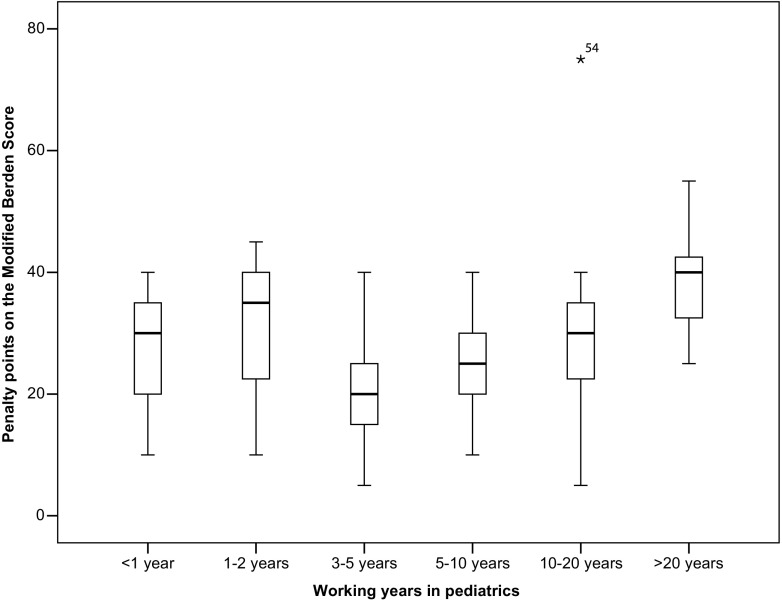
Association between years of experience in pediatrics and practical test result. Significant difference amongst groups (*p* < 0.05), with an optimum of PBLS skills in participants with 3–5 years of working experience

**Fig. 4:**
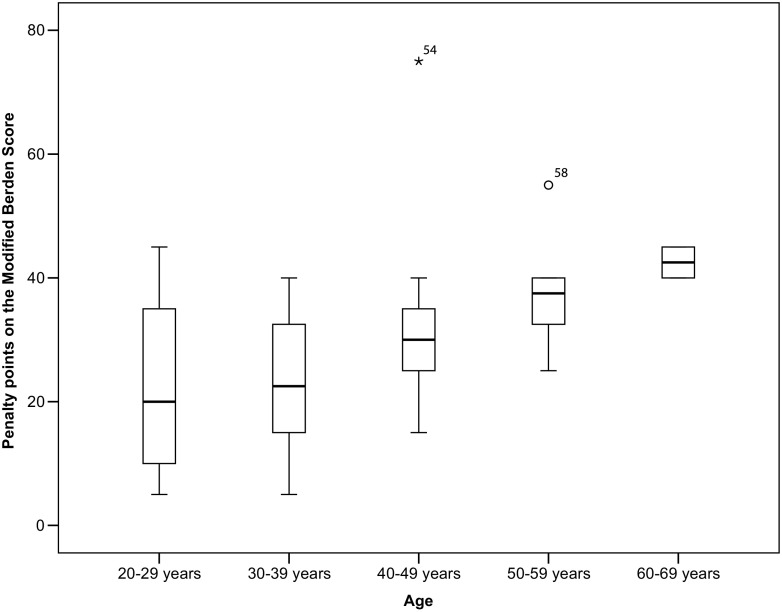
Association between age and practical test result. Significant difference amongst groups (*p* < 0.05), with a decline in PBLS skills with increasing age

The penalty score on the practical exam was not affected by sex, frequency of PBLS courses, being PBLS instructor, type of hospital, and number of witnessed IHCAs. None of the participants had witnessed an OHCA. Participants who were older, attending, and more experienced in terms of years working in pediatrics had higher penalty scores than their younger colleagues, who had less experience in pediatrics (Figs. 3 and 4, Table 3). There was no significant association between age and time since last PBLS course.

**Table 3:** Participant characteristics associated with pediatric basic life support skills

Characteristic	Subgroups	Mean penalty points	Number	*p* value
Sex	Male	28.6	19	0.55
	Female	27.4	39	
Age	20–29 years	23.6	14	0.01*
	30-39 years	23.5	20	
	40–49 years	31.1	14	
	50–59 years	37.5	8	
	60–69 years	42.5	2	
Hospital type	General	29.0	35	0.17
	Academic	26.3	23	
Specialization level	Resident	22.1	24	0.00*
	Attending	32.1	34	
Years of experience in pediatrics	< 1 year	27.0	5	0.01*
	1–2 years	30.7	7	
	3–5 years	19.7	16	
	5–10 years	25.0	6	
	10–20 years	30.9	16	
	> 20 years	38.8	8	
Frequency of PBLS courses	Every 3 months	32.5	6	0.30
	Every 6 months	30.0	6	
	Every 12 months	26.4	29	
	Less than once a year	18.8	4	
	Never	31.2	13	
Last PBLS course	< 3 months ago	24.2	19	0.11
	3–6 months ago	23.5	10	
	6–12 months ago	30.0	14	
	1–2 years ago	29.0	10	
	> 2 years ago	43.0	5	
(P)BLS/PALS instructor	Yes	20.0	7	0.07
	No	29.0	51	
Witnessed IHCA	≤ 5 times	26.6	42	0.25
	> 5 times	28.1	16	

*(P)BLS* (pediatric) basic life support, *PALS* Pediatric Advanced Life Support, *IHCA* in-hospital cardiac arrest

*Significant difference amongst groups (*p* < 0.05)

Fifty-one percent of our participants considered themselves competent in PBLS. No correlation was found between self-assessed competence and performance on the practical exam (Spearman’s rho − 0.154, *p* = 0.25).

## Discussion

Retention of PBLS skills amongst pediatricians and residents was discouraging, with a pass rate of 21% on the unannounced practical exam. Compressions/ventilations were slightly better performed than other parts of the algorithm. Numerous participants (29%) failed to use the correct compression-ventilation ratio. Most often, the adult ratio of 30:2 was applied. According to the ERC guidelines, lay people, only trained in adult BLS, may use this ratio in the resuscitation of children [8, 16]. However, since asphyxial etiologies of cardiac arrest are more prevalent in children, medical professionals trained in PBLS are expected to resuscitate children with a ratio of 15:2. A few senior pediatricians used a ratio of 5:1, which indicates that they were not aware of guideline updates [1, 2, 16]. Also, participants sometimes seemed to compensate inadequate ventilations, thus delivering ≥ 3 ventilations per cycle.

Theoretical knowledge was retained better than practical skills, which is consistent with existing literature [5, 7, 22, 27]. Pediatricians and residents are perhaps more exposed to theoretical aspects of PBLS through case discussions, observing simulated or real-life resuscitations, and studying CPR textbooks and resuscitation guidelines, than to hands-on practice. The focus of various instructional methods, such as e-learnings, lies on theoretical knowledge. In our experience, simulation training often involves Pediatric Advanced Life Support (PALS) scenarios with a focus on systematic (ABCDE) assessment, medication and equipment, clinical reasoning, and teamwork, instead of PBLS with pure emphasis on the quality of compressions and ventilations. Refresher courses/booster training sessions with sufficient hands-on practice to rehearse compressions and (mouth-to-mouth) ventilations should therefore be encouraged.

Our findings are in agreement with previous studies that documented poor retention of skills amongst healthcare professionals [9, 13, 17, 18, 23, 25]. We found a less clear-cut association between last PBLS course and practical skills, though. This might be due to our limited number of participants. Previous studies on retention of resuscitation skills, that used *scheduled* testing, included 19–224 (mean 84) participants [5, 7, 13, 20, 22, 26–28]. One of the scarce studies that used unannounced testing of resuscitation skills included 49 participants [29]. Information on the exact time of last PBLS course was obtained by participant self-reporting, possibly introducing recall bias. Furthermore, several pediatricians indicated that they also attended adult BLS training. This could have skewed our results in opposite directions. On the one hand, various items in adult BLS correspond with PBLS, so this training can be regarded as a partial refresher for PBLS. Conversely, some fundamental differences exist between adult BLS and PBLS, such as the compression-ventilation ratio and five initial rescue breaths. Participants that recently attended adult BLS training might have erroneously applied the adult ratio, as we discussed previously. We did not inquire after adult BLS training in our questionnaire, but were informed about this by some participants.

Three variables were significantly and inversely related to PBLS skills: age, years of experience in pediatrics, and specialization level. In contrast with previous studies [5, 20, 26], we did not find a direct association between frequency of PBLS courses and retention of skills. Nevertheless, the importance of frequent PBLS training might still indirectly become clear. That is, residents performed better than attendings on the practical exam. An explanation could be that residents follow more frequent PBLS training (in addition to official courses) as part of their medical education than attendings and therefore retain their PBLS skills better. None of the participants had witnessed an OHCA. Only 21% of the participants had witnessed > 10 IHCAs. Evidently, there are limited clinical opportunities for pediatric professionals to consolidate their resuscitation skills. More working years in pediatrics does therefore not automatically lead to more competence in PBLS.

Approximately half of our participants considered themselves proficient in PBLS. This is comparable with previous studies, in which ~ 30–50% of the participants felt sufficiently qualified to conduct (pediatric) life support [6, 10, 30]. Many studies in this respect pertained to medical students or residents, whereas we also included attending physicians. During our unannounced visits, several pediatricians indicated that they were not available for testing. This may have led to the inclusion of pediatricians who felt more equipped to perform PBLS, possibly resulting in a higher level of self-assessed competence. Participants may have also overestimated themselves, because their self-assessed competence did not correlate with their performance on the practical exam. This is an important consideration in light of the finding that people who overestimate themselves are probably less susceptible to corrective feedback [24].

A study design in which participants would be recruited and examined at different time intervals from last certification was considered as an alternative strategy to ours. However, such a design entails a major logistic challenge and does not really allow for unannounced testing. Moreover, time intervals in our study are automatically varied, because participants attended their last PBLS course at different moments in the past. In addition, it would have been informative to compare our (practical) exam results with the scores of the participants on their last PBLS certification. However, the latter scores were confidential, unavailable, and incomparable to ours, for we used a new, validated scoring tool to assess PBLS skills. Although exact pass rates could not be provided, the Dutch Foundation for the Emergency Medical Care of Children affirmed that almost all candidates pass the PBLS exam at the end of the course, which argues against poor acquisition of skills.

## Strengths

Our main strength was the unannounced examination. Truly unannounced testing without any prior notification was ethically and logistically impossible. Most importantly, participants were unable to prepare themselves for the ad hoc exam, thereby creating a situation that closely resembles real-life pediatric resuscitations. By contrast, in many previous studies and conventional PBLS exams, candidates are examined at a predefined moment (after training).

We used a valid and reliable scoring instrument for the practical exam. We meticulously composed a MCQ to assess PBLS knowledge. A validated MCQ was not available in The Netherlands. We considered using a standard theoretical PBLS test from abroad. However, even if a *valid and reliable* theoretical PBLS test, based on European guidelines, could be found, it would have required a separate study to validate a translated version of this test for the Dutch situation. Our MCQ had content validity, because it was based on the 2015 ERC guidelines [16]. Through critical appraisal by experts, it also gained face validity. The MCQ had good feasibility, for it could be completed in 2–3 min. Most answers to the MCQ questions could not be readily derived from the practical exam.

## Limitations

We used a non-recording manikin, because such lower fidelity manikins are still widely used, both in The Netherlands and abroad (Laerdal Corporation, personal communication), and because our validated scoring instrument was developed for these manikins. The ERC considers the use of lower fidelity manikins appropriate for all levels of training [8]. Moreover, the only skill guide available for the Resusci® Junior Basic until now does not generate quantitative, storable data, and does not provide feedback on all aspects of compressions and ventilations.

Instead of using video recordings, PBLS skills were scored by direct visual assessment. Video-based assessment has some advantages, but also some audiovisual shortcomings. As Jones et al. state: ‘The perspective of the camera is unlike the human eye, and does not have the same peripheral and depth perception that humans have” [12]. Considerations of the candidates may also be inaudible on video. We believe that video-based assessment is not necessarily superior to direct visual assessment, provided that two examiners are involved in the latter, which was the case in this study. One examiner leveled to the manikin’s chest to observe compressions and ventilations from the side of the manikin, as suggested by Jones et al. [12]. The other examiner recorded the duration of compressions and ventilations. It has been reported that most clinicians do not use CPR feedback devices and rely on visual assessment to assess CPR quality during clinical care [12]. As long as this happens on a large scale in real life, it seemed justifiable to assess PBLS visually in this study.

We may have induced selection bias, since pediatricians/residents could decline participation at the time of our visit. The ones who felt more competent may have been more inclined to take part. If so, the pass rates in our study are an overestimation of the true PBLS knowledge and skills amongst pediatricians/residents. This only corroborates the contention that retention of PBLS skills requires attention. In spite of this speculation, we believe that most pediatricians/residents were truly too busy to participate. We were unfortunately not informed about the exact number of pediatricians/residents that were present, but not available for testing during our visit.

Younger participants performed PBLS skills significantly better than their senior colleagues. Since we included more younger participants (34 participants < 40 years vs. 24 participants > 40 years), the skewness in age might have led to an overestimation of PBLS skills. Again, this would underscore our claim that the resuscitation preparedness of pediatricians needs attention.

Finally, we used a five-point Likert scale for self-assessment. Such a categorical scale has been used for self-assessment of resuscitation skills before [24]. We realize, however, that this reduced the precision of our measurement and may have prevented us from finding a correlation between self-assessed competence and performance on the practical exam.

## Conclusions

Retention of PBLS skills appears to be poor amongst pediatricians and residents. PBLS knowledge is retained a little better. More research is needed to identify ways to ameliorate the resuscitation preparedness of pediatricians and residents with the ultimate goal to improve the survival rate of children with cardiac arrest.

## Electronic supplementary material

ESM 1 Theoretical test (MCQ) used in this study. (PDF 111kb)

## Abbreviations

AED: Automated external defibrillator
AHA: American Heart Association
CPR: Cardiopulmonary resuscitation
EPALS: European Pediatric Advanced Life Support
ERC: European Resuscitation Council
IHCA: In-hospital cardiac arrest
MCQ: Multiple-choice test
OHCA: Out-of-hospital cardiac arrest
PALS: Pediatric Advanced Life Support
PBLS: Pediatric basic life support

## References

[1] (2000) Guidelines 2000 for Cardiopulmonary Resuscitation and Emergency Cardiovascular Care. Part 9: pediatric basic life support. The American Heart Association in collaboration with the International Liaison Committee on Resuscitation. Circulation 102(8 Suppl):I253–I290 10966678

[2] Atkins DL, Berger S, Duff JP, Gonzales JC, Hunt EA, Joyner BL, Meaney PA, Niles DE, Samson RA, Schexnayder SM (2015). Part 11: pediatric basic life support and cardiopulmonary resuscitation quality: 2015 American Heart Association guidelines update for cardiopulmonary resuscitation and emergency cardiovascular care. Circulation.

[3] Bardai A, Berdowski J, van der Werf C, Blom MT, Ceelen M, van Langen IM, Tijssen JG, Wilde AA, Koster RW, Tan HL (2011). Incidence, causes, and outcomes of out-of-hospital cardiac arrest in children. A comprehensive, prospective, population-based study in the Netherlands. J Am Coll Cardiol.

[4] Berden HJ, Pijls NH, Willems FF, Hendrick JM, Crul JF (1992). A scoring system for basic cardiac life support skills in training situations. Resuscitation.

[5] Broomfield R (1996). A quasi-experimental research to investigate the retention of basic cardiopulmonary resuscitation skills and knowledge by qualified nurses following a course in professional development. J Adv Nurs.

[6] Freund Y, Duchateau FX, Baker EC, Goulet H, Carreira S, Schmidt M, Riou B, Rouby JJ, Duguet A (2013). Self-perception of knowledge and confidence in performing basic life support among medical students. Eur J Emerg Med.

[7] Gass DA, Curry L (1983). Physicians’ and nurses’ retention of knowledge and skill after training in cardiopulmonary resuscitation. Can Med Assoc J.

[8] Greif R, Lockey AS, Conaghan P, Lippert A, De Vries W, Monsieurs KG, Education and implementation of resuscitation section Collaborators; Collaborators (2015). European Resuscitation Council Guidelines for Resuscitation 2015: Section 10. Education and implementation of resuscitation. Resuscitation.

[9] Hamilton R (2005). Nurses’ knowledge and skill retention following cardiopulmonary resuscitation training: a review of the literature. J Adv Nurs.

[10] Hayes CW, Rhee A, Detsky ME, Leblanc VR, Wax RS (2007). Residents feel unprepared and unsupervised as leaders of cardiac arrest teams in teaching hospitals: a survey of internal medicine residents. Crit Care Med.

[11] Jewkes F, Phillips B (2003). Resuscitation training of paediatricians. Arch Dis Child.

[12] Jones A, Lin Y, Nettel-Aguirre A, Gilfoyle E, Cheng A (2015). Visual assessment of CPR quality during pediatric cardiac arrest: does point of view matter?. Resuscitation.

[13] Kaye W, Mancini ME (1986). Retention of cardiopulmonary resuscitation skills by physicians, registered nurses, and the general public. Crit Care Med.

[14] Kramer-Johansen J, Myklebust H, Wik L, Fellows B, Svensson L, Sørebø H, Steen PA (2006). Quality of out-of-hospital cardiopulmonary resuscitation with real time automated feedback: a prospective interventional study. Resuscitation.

[15] Mac Giolla Phadraig C, Ho JD, Guerin S, Yeoh YL, Mohamed Medhat M, Doody K, Hwang S, Hania M, Boggs S, Nolan A, Nunn J (2016). Neither Basic Life Support knowledge nor self-efficacy are predictive of skills among dental students. Eur J Dent Educ.

[16] Maconochie IK, Bingham R, Eich C, López-Herce J, Rodríguez-Núñez A, Rajka T, Van de Voorde P, Zideman DA, Biarent D (2015). European resuscitation council guidelines for resuscitation 2015 section 6. Paediatric life support. Resuscitation.

[17] Madden C (2006). Undergraduate nursing students’ acquisition and retention of CPR knowledge and skills. Nurse Educ Today.

[18] Mancini ME, Kaye W (1985). The effect of time since training on house officers’ retention of cardiopulmonary resuscitation skills. Am J Emerg Med.

[19] Moser DK, Coleman S (1992). Recommendations for improving cardiopulmonary resuscitation skills retention. Heart Lung.

[20] Na JU, Sim MS, Jo IJ, Song HG, Song KJ (2012). Basic life support skill retention of medical interns and the effect of clinical experience of cardiopulmonary resuscitation. Emerg Med J.

[21] Niles D, Sutton RM, Donoghue A, Kalsi MS, Roberts K, Boyle L, Nishisaki A, Arbogast KB, Helfaer M, Nadkarni V (2009). “Rolling Refreshers”: a novel approach to maintain CPR psychomotor skill competence. Resuscitation.

[22] O’Steen DS, Kee CC, Minick MP (1996). The retention of advanced cardiac life support knowledge among registered nurses. J Nurses Staff Dev.

[23] Patel J, Posencheg M, Ades A (2012). Proficiency and retention of neonatal resuscitation skills by pediatric residents. Pediatrics.

[24] Plant JL, Corden M, Mourad M, O’Brien BC, Schaik SM (2013). Understanding self-assessment as an informed process: residents’ use of external information for self-assessment of performance in simulated resuscitations. Adv Health Sci Educ.

[25] Semeraro F, Signore L, Cerchiari EL (2006). Retention of CPR performance in anaesthetists. Resuscitation.

[26] Seraj MA, Naguib M (1990). Cardiopulmonary resuscitation skills of medical professionals. Resuscitation.

[27] Smith KK, Gilcreast D, Pierce K (2008). Evaluation of staff's retention of ACLS and BLS skills. Resuscitation.

[28] Sutton RM, Niles D, Meaney PA, Aplenc R, French B, Abella BS, Lengetti EL, Berg RA, Helfaer MA, Nadkarni V (2011). Low-dose, high-frequency CPR training improves skill retention of in-hospital pediatric providers. Pediatrics.

[29] Turner NM, Lukkassen I, Bakker N, Draaisma J, ten Cate OT (2009). The effect of the APLS-course on self-efficacy and its relationship to behavioral decisions in pediatric resuscitation. Resuscitation.

[30] Van Schaik SM, Von Kohorn I, O’Sullivan P (2008). Pediatric resident confidence in resuscitation skills relates to mock code experience. Clin Pediatr.

