# Supplementary material for: Retention of knowledge and skills in pediatric basic life support amongst pediatricians
Source: Eur J Pediatr. 2018 May 7;177(7):1089–99. doi: 10.1007/s00431-018-3161-7 (PMC5997099; doi:10.1007/s00431-018-3161-7)
Supplement: Supplementary file 1 — Theoretical test (MCQ) used in this study. (PDF 111kb) [file 431_2018_3161_MOESM1_ESM.pdf]

Theoretical exam

Explanation

The questions below pertain to the *basic life support* in children (age: 1 month to adolescence). These questions are based on the European guideline for basic life support (BLS) in children, as formulated by the European Resuscitation Council (ERC) in 2010 and 2015. There will be no correction for guessing on this multiple-choice test. You will pass this test if ≥ 8 questions are answered correctly.

Instructions

Please select the correct answer for each question. Each question has only 1 correct answer.

Questions

1. What is the best criterion to determine the need for chest compressions in a child?
   - ☐ The absence of arterial pulsations
   - ☐ The absence of signs of life

2. In which position should an infant's (<1 year) head be held during mouth-to-mouth ventilations?
   - ☐ Neutral position
   - ☐ Head tilt ("sniffing position")

3. What is the maximum time interval to assess breathing in an unwell child using the look-listen-feel technique?
   - ☐ 5 seconds
   - ☐ 10 seconds
   - ☐ 20 seconds
   - ☐ 30 seconds

4. Between which minimum and maximum should the chest compressions rate be kept during the resuscitation of a child?
   - ☐ 100 and 120 compressions per minute, respectively
   - ☐ 100 and 140 compressions per minute, respectively
   - ☐ 80 and 120 compressions per minute, respectively
   - ☐ 60 and 120 compressions per minute, respectively

5. When performing chest compressions on a child, the sternum must be pressed down to at least:
   - ☐ 1/4 of the anterior-to-posterior thoracic diameter
   - ☐ 1/3 of the anterior-to-posterior thoracic diameter
   - ☐ 2/3 of the anterior-to-posterior thoracic diameter
   - ☐ 1/2 of the anterior-to-posterior thoracic diameter

6. In children, a primary cardiac arrest due to cardiac arrhythmia is more common than a secondary cardiac arrest.
   - ☐ Correct
   - ☐ Incorrect

7. What is the recommended energy level for defibrillation of a child with a manual defibrillator?
   - ☐ 1 Joule per kilogram body weight
   - ☐ 2 Joules per kilogram body weight
   - ☐ 4 Joules per kilogram body weight
   - ☐ 150 Joules

8. When a chocking, conscious child turns red and starts coughing with deep inhaling breaths in between, the child must be stimulated to continue coughing. You do not have to intervene otherwise as long as the situation does not deteriorate.
   - ☐ Correct
   - ☐ Incorrect

9. In a child, the recommended compression-to-ventilation ratio for professionals is:
   - ☐ 30:2
   - ☐ 15:5
   - ☐ 15:2
   - ☐ 3:1

10. When you witness the sudden collapse of a child and you are the only person around, you should:
    - ☐ First call for help and try to get an AED
    - ☐ First start resuscitation (BLS) for 1 minute before getting help and/or getting an AED
    - ☐ First give 5 rescue breaths

------ End of theoretical exam------
